# Supplementary material for: Are carbon emissions trading and green financial instruments synergistic? -Comprehensive quantitative research based on content analysis
Source: PLoS One. 2024 Mar 7;19(3):e0298601. doi: 10.1371/journal.pone.0298601 (PMC10919601; doi:10.1371/journal.pone.0298601)
Supplement: S1 Data — (ZIP) [file pone.0298601.s001.zip › Data/地级市Z_F工作报告.zip!/░▓╗╒/┴∙░▓╩╨/└┤╫╘╣1⁄2╓┌║┼í╛┬φ┐╦╩2╛▌═°í┐_2018.docx]

各位代表：

现在，我代表市人民政府，向大会报告政府工作，请予审议,并请市政协委员和其他列席人员提出意见。

一、坚持红色土地绿色振兴，六安发展进入新时代

市四届人大一次会议以来的四年，是六安发展进程中极不平凡的四年。全市人民在省委、省政府和市委的坚强领导下，全面贯彻党的十八大、十九大精神和习近平总书记视察六安重要讲话精神，以习近平新时代中国特色社会主义思想为指导，全面落实市第三次、第四次党代会决策部署，攻坚克难，真抓实干，胜利完成“十二五”规划，顺利实施“十三五”规划，保持了经济平稳较快健康发展和社会和谐稳定。

**——综合实力跃上新台阶。**全面贯彻新发展理念，扎实推进供给侧结构性改革，着力做大总量、提升质量。生产总值突破千亿元，预计达1240亿元、年均增长7.5%；财政收入达184亿元、年均增长11.3%；固定资产投资达1204亿元、年均增长12.9%。产业结构不断优化，三次产业比例由2013年的18.2:49.8:32调整为15.2:45.4:39.4。粮食生产连年丰收，农业产业化步伐加快，荣获全国粮食生产先进市和全省现代生态农业产业化示范市。累计完成工业技改投资近千亿元，规模工业增加值年均增长7.5%。社会消费品零售总额达604亿元、年均增长11.7%。

**——绿色振兴开启新局面。** “一心一廊”“一谷一带”“一岭一库”建设扎实推进，合六叶工业走廊对全市工业增长贡献率达55.5%；六安茶谷园区获评全国六安瓜片茶产业知名品牌示范区。科技创新能力不断增强，累计发明专利有效量986件、年均增长68%，高新技术产业增加值年均增长10.8%，首获全国科技进步先进市；战略性新兴产业产值占规上工业总产值比重达到20%。省级战新基地和新兴产业重大工程、重大专项实现零的突破，霍邱县、霍山县、叶集区经济开发区被列为全国循环化改造示范试点园区。旅游业综合收入年均增长28.1%，全市5A 级景区2家、4A级景区25家，居全省前列，荣获全国森林旅游示范市。环境保护进一步加强，主要污染物排放量持续消减，大气和水环境质量保持全省前列。2016年全市生态系统生产总值超过2500亿元，被列为国家级生态示范区、生态环境监察试点市、环保部绿色GDP2.0试点市、全国低碳试点城市。霍山县、舒城县成为国家生态县，金寨县成功创建全国首批生态文明示范县。

**——改革开放取得新突破。**坚持围绕发展抓改革。金寨县农村土地制度“三项改革”等一批“国字号”改革试点示范成效显著。农村土地确权登记颁证工作全省领先，“三变”改革试点全面推进，市县乡村四级“河长制”全面建立。“放管服”改革纵深推进，市级行政审批事项精简至168项，“3+2”清单制度体系实现全覆盖。商事制度改革不断深化，“多证合一”“一照一码”改革全面实施，各类市场主体由15.8万户增至24.2万户。叶集经国务院批准成为市辖行政区。国资国企改革、事业单位转企改制、财税改革取得实质性进展。地方金融改革成效明显，农村信用联社转制为法人银行，村镇银行实现县区全覆盖。医药卫生体制综合改革取得突破，基本医疗卫生事业的公益性不断强化。完成公务用车改革和市辖城区公交、出租车运营体制改革。坚持围绕发展抓开放。大力开展招商引资，累计到位内资2025.9亿元、年均增长12.4%，利用外资14.8亿美元、年均增长9.6%；累计引进10亿元以上项目73个，上市公司、500强、央企投资项目106个。推进外贸优进优出，累计实现进出口总额24.7亿美元。国际合作取得进展，与国外2个城市结为友好城市、与2个城市发展为友好合作关系。坚持打造节会大平台，扩大六安知名度美誉度，成功举办四届大别山（六安）山水文化旅游节、两届中国（六安）国际羽绒博览会、两次大别山革命老区鄂豫皖三省政协主席座谈会。

**——城乡面貌发生重大变化。**大力实施新型城镇化带动战略，城乡规划体系不断完善，在全省率先完成各县总规修编。常住人口城镇化率由40.4%提高到45%。中心城市建设步伐加快，累计实施重点工程130个、完成政府性投资180亿元，建成区面积扩展到76平方公里，荣获国家园林城市、国家森林城市、国家节水型城市、中国人居环境奖、“魅力中国城”十佳城市。各县县城建设框架全面拉开，基础设施进一步改善；特色小镇建设亮点纷呈，裕安区独山镇、金安区毛坦厂镇荣膺全国特色小镇，10个镇入选全国重点镇；349个省级美丽乡村中心村建成，“三线三边”城乡环境整治成效显著。交通实现跨越式发展，阜六铁路、宁西铁路复线等建成通车，成为横贯东西、纵达南北的节点城市；大别山旅游扶贫快速通道全线贯通，312国道六安段、六舒三路升级改造完成，新改建国省干线1000公里，建设里程是前12年的总和；农村公路加快建设，市县快速通达工程全面建成，全市公路总里程居全省首位，被确定为国家公路运输枢纽城市。淠河、史河治理等重大水利工程基本建成。高压输变电工程和农村电网改造快速推进，完成投资26亿元。

**——脱贫攻坚实现重大进展。**始终把脱贫攻坚作为头等大事和第一民生工程，深入推进精准扶贫精准脱贫。累计实现257个贫困村出列、48万贫困人口脱贫，贫困发生率从13.84%下降至4.5%左右。坚持精准识别、动态管理，对建档立卡工作开展了“回头看”和精准核查，确保扶贫对象真实准确。大力实施脱贫攻坚“十大工程”，推广“四带一自”产业扶贫模式，实现产业扶贫全覆盖，累计带动18.5万贫困户发展特色种养业，建成村级光伏电站692个、户用光伏电站5.82万个，金寨县光伏扶贫“三阶段、四模式”做法得到中央领导充分肯定；建设村级电商服务网点近3000个，建成就业扶贫驿站42个；认真落实“351”“180”医疗保障政策，在全省率先实现城镇居民医保和新农合两项制度“并轨”，本地常住贫困人口家庭医生签约率达100%。加快易地扶贫搬迁，累计完成2.04万人搬迁任务。强化资金保障，累计投入各类扶贫资金64亿元。压紧压实各级责任，设立“四级五支队伍”。深入实施结对帮扶、“百企帮百村”、“扶贫日”等活动，大扶贫格局日益形成。

**——人民群众获得感不断提升。**预计城镇、农村常住居民年人均可支配收入达2.67万元和1.09万元，年均增长8.3%和9.7%。加强普惠性、基础性、兜底性民生建设，坚持每年在实施省33项民生工程基础上，累计增加实施8项市级项目，民生工程财政投入资金359.5亿元、年均增长11.3%，民生工程实施工作连年全省领先。就业形势总体稳定，城镇累计新增就业29.2万人。社会保障制度实现全覆盖，大病和应急救助制度不断完善，市残疾人康复中心建成使用。建设各类保障性安居工程11.7万套，改造农村危房6.7万户。组织实施两轮学前教育、两轮城区义务段教育学校建设三年行动计划、改善农村义务教育薄弱学校五年规划，推进新一轮普通高中布局调整，提前两年实现国家义务教育发展基本均衡县（区）全覆盖。中国特色社会主义和中国梦深入人心，26人荣登“中国好人榜”，5人获全国道德模范提名，荣获第四届省文明城市。文化事业和文化产业蓬勃发展，公共文化服务水平不断提高。成功举办国际马拉松赛、全国登山大会和市第四届运动会。

**——社会和谐稳定局面不断巩固。**健全完善社会治理体系，改革社会组织管理制度。加强社区依法自治和协商民主制度建设，推进乡镇、村（社区）为民服务中心标准化，实施为民服务全程代理、“一站式”服务。深入推进平安六安建设，完善公共安全防控体系，全市刑事案件总数、八类严重暴力犯罪、侵财类案件逐年下降，命案破案率100%，人民群众安全感大幅提升。成功处置“3•11”持枪劫持人质案件。全面加强生产安全、食品药品安全、信息网络安全建设，有效预防和遏制较大以上事故发生。完成“六五”普法，启动“七五”普法。全力打造阳光信访、法治信访、责任信访，信访秩序稳中向好，圆满完成党的十九大期间信访维稳安保任务。全面加强民族宗教工作，促进民族团结宗教和谐。积极支持国防和军队改革建设，聚力推进军民融合深度发展，国防动员、国防教育、兵役征集、人民防空工作扎实推进，第三次荣获全国双拥模范城。广播电视、新闻出版、档案、地方志、对台、老龄、工会、共青团、全民科学素质、妇女儿童、社会慈善等事业取得新成绩。

**——政府自身建设不断加强。**大力推进法治政府建设，建立健全政府法律顾问制度和重大决策合法性审查机制。不断规范行政执法行为，强化执法监督，提高行政执法水平。出台《关于进一步加强与市人大市政协工作联系的意见》，实现自觉接受人大依法监督、政协民主监督制度化；累计办理人大议案8件、代表建议538件和政协委员提案1112件，办理水平不断提升。大力推进效能建设，健全抓落实工作机制，政府系统执行力不断增强。“互联网+政务服务”工作暨智慧政务建设加快推进，政务公开广度和深度进一步拓展。扎实推进政府系统全面从严治党，严格落实“一岗双责”，全面加强作风建设和廉政建设，推进巡察监督和审计监督全覆盖，营造了风清气正、干事创业的良好政治生态。

刚刚过去的2017年，我们坚持以提高发展质量效益为中心，以推进供给侧结构性改革为主线，以实施五大发展行动计划为抓手，顺利完成市四届人大四次会议确定的各项目标任务，主要经济指标好于预期、高于去年同期，社会大局和谐稳定。预计生产总值增长8%左右，高于年度计划0.5个百分点；财政收入增长20%，高于年度计划11.5个百分点；城镇、农村常住居民人均可支配收入分别增长8%、9%。**一是稳增长、调结构。**全面落实中央、省稳增长各项政策措施，深入开展“暖企”行动和“四送一服”双千工程，预计规模工业增加值增长8%；工业用电量保持全省前列，新增国家智能制造试点示范项目1个、安徽工业精品6项。处置“僵尸企业”80家，盘活闲置土地1.5万亩；商品房去化周期22.7个月，较上年减少4.6个月，其中住宅去化周期16.3个月、较上年减少2.3个月；改善实体经济金融供给，预计全年新增贷款337亿元、增长66.4%，增量和增幅均居全省前列。推动项目提质增效，预计固定资产投资增长12%；沃特玛、宏芯集成电路、悠然南山二期、华润电力（霍山）气光能源互补等195个项目开工建设；完成国省干线投资36.1亿元、水利建设投资9.7亿元。加快农业现代化建设，粮食总产315.8万吨，5个农业产业园区入选全国农村创业创新园区（基地）目录，六安瓜片获评中国十大茶叶区域公用品牌，霍山黄芽获列中欧地理标志产品互认互保“100+100”公示清单。**二是重创新、优生态。**大力实施创新驱动，建成科技企业孵化器7家、省级备案众创空间5家，9个项目获批省级重大科技专项。六安大学科技园核心区一期投入使用。大力推进“三重一创”建设，1-11月份，高新技术产业增加值增长21.2%、增幅居全省第四，战新产业产值增长27.6%，舒城杭埠经济开发区入选省级电子信息产业基地。促进消费升级，预计社会消费品零售额增长11.5%。实施重点旅游项目146个、总投资138.5亿元，天堂寨列入国家生态旅游示范区、仙人冲画家村入围全国乡村旅游创客示范基地，成功注册“大别山风景道”商标。全面打响大气、水、土壤污染防治攻坚战，强力推进中央和省环保督察反馈问题整改，空气质量优良率81.4%，地表水考核断面全部达标。**三是推改革、促开放。**开展相对集中行政许可权改革试点，金寨县在全省率先组建行政审批局并挂牌运行。全面完成农村土地确权登记颁证试点工作，确权率100%。推进生态保护红线划定，完成永久基本农田全域划定。基本完成市级层面户籍制度改革。招商引资实现突破，聚焦IPO绿色通道精准招商，54家企业完成注册、总投资242亿元。预计实现到位内资500亿元、增长12.4%，直接利用外资4.18亿美元、增长10%。对外贸易持续增长，预计实现进出口总额7亿美元、增长32%。深化区域合作，积极推进合六市郊铁路、合六南通道、合六共建园区等一批重点项目。**四是统城乡、强协调。**加快中心城区提质扩容，实施重点工程82个、完成政府性投资47.2亿元，六安火车站新站房主体工程完工。积极开展特色镇村培育，霍山县上土市温泉小镇获批第一批省级特色小镇。扎实开展47个乡镇政府驻地整治建设和118个省级中心村、95个县区自主建设中心村建设，裕安区、金寨县、舒城县荣获全省美丽乡村建设先进县。深入推进农村垃圾、污水、厕所专项整治“三大革命”，荣获全国厕所革命优秀城市奖。**五是抓脱贫、惠民生。**坚决打好脱贫攻坚年度战役，扎实开展“重精准、补短板、促攻坚”专项整改行动，推进精准扶贫、监测评估、防范返贫、责任落实机制全覆盖，预计出列贫困村228个，减少贫困人口13万人以上，脱贫攻坚再战再捷。精心组织实施民生工程，投入财政资金108.1亿元。城镇新增就业4.96万人，完成率183.7%；城乡居民基本医疗保险参保532.3万人，超额完成目标任务。城乡居民大病保险补偿等政策全部落实，医保即时结算服务体系建成。加快县域医共体建设，促进县乡村三级联动发展，16所乡镇卫生院获国家群众满意乡镇卫生院称号。推动文化改革发展，开展文化科技卫生“三下乡”“书香六安•悦读人生”全民阅读等文化活动，多部文艺作品获省级以上重要奖项；市图书档案馆、科技文化馆基本建成。

各位代表！过去四年，我们收获了太多激动和感慨，特别是习近平总书记2016年亲临视察，为我们发展指明了前进方向、提供了根本遵循；全市上下心齐劲足、拧成一股绳，自觉投身到幸福六安建设的伟大事业中，皖西大地日新月异、欣欣向荣，呈现出风清气正、团结和谐、蓬勃向上的生动局面。过去的四年，是全市经济社会发展取得重大进展的四年，是综合竞争力和对外影响力大幅提升的四年，是人民群众获得感最强的四年。这些成绩的取得，饱含着党中央、国务院和习近平总书记对革命老区的亲切关怀，凝聚着省委、省政府对六安发展的关心支持，得益于市委的坚强领导、科学决策以及市人大和市政协的监督支持、社会各界的悉心帮助，归功于党的理论路线方针政策的真理力量和全市干部群众的不懈奋斗。在此，我谨代表市人民政府，向大家表示诚挚的感谢！

各位代表、同志们！我们在为过去四年取得的巨大成就感到自豪的同时，还必须清醒地看到，我市人口数量全省靠前、经济总量居中、人均水平偏后的现状没有改变，发展不平衡不充分的问题十分突出，脱贫攻坚任务艰巨，生态环境保护任重道远；工业化、城镇化水平较低，农业农村基础依然薄弱，县域经济发展面临新的困难；新旧动能转换不快，创新能力不强，科技、人才、资金等要素瓶颈制约依然突出；实体经济困难较多，民间投资增长乏力；民生领域还有不少短板，公共服务供给不足；法治政府建设任务依然繁重，政府效能有待提升，干部作风建设永远在路上。问题所在是施政所向，我们一定高度重视，着力加以解决。

二、决胜全面建成小康社会，开启新时代幸福六安建设新征程

党的十九大提出了全面建设社会主义现代化强国的宏伟目标，开启了中国特色社会主义建设的新征程。市委作出了“三个同步”的战略部署。站在新的历史起点，今后五年，我们要确保到2020年与全省全国同步全面建成小康社会，要为到2035年与全省全国同步基本实现社会主义现代化、到本世纪中叶与全省全国同步实现社会主义现代化打下坚实基础。使命光荣而艰巨，责任重大而神圣！

今后五年工作的总体思路是：全面贯彻党的十九大精神，以习近平新时代中国特色社会主义思想为指导，统筹推进“五位一体”总体布局，协调推进“四个全面”战略布局，紧扣社会主要矛盾新变化，坚定不移践行新发展理念，坚定不移坚持稳中求进工作总基调，坚定不移锁定绿色振兴主取向，坚定不移激发改革创新主动力，坚定不移打好防范化解重大风险、精准脱贫、污染防治攻坚战，沿着高质量发展之路开拓进取，奋力书写新时代老区振兴辉煌篇章。

今后五年全市经济社会发展的主要目标是：

**——综合实力明显提升。**经济保持中高速增长，财政收入迈过300亿元。农业综合实力跃居全省第一方阵；规模以上工业增加值增幅高于经济增长速度，打造产值百亿级企业4－5家、千亿级产业园区1－2个；服务业发展与全省同步。建成安徽省农业一二三产融合发展基地、铁矿资源精深加工基地、生态休闲旅游度假目的地和大别山革命老区核心增长极。

**——产业结构明显优化。**一产比重明显下降，二三产业比重持续上升。战略性新兴产业产值占规上工业总产值比重达到30%以上。现代服务业增加值占服务业比重提高到 40%。科技进步贡献率、全要素生产率显著提升，产业转型升级步伐加快。

**——绿色效应明显增强。**绿色发展理念深入人心，绿色产业体系特色彰显，大别山绿色发展示范区建设取得明显成效，勇当全省绿色发展排头兵。主要环境指标处于全省领先水平，森林覆盖率、主要河湖水质优良比例进一步提高，主要污染物排放总量持续下降，全域黑臭水体全部消除。

**——城乡发展更加协调。**全市常住人口城镇化率提高到50%以上。大别山区域中心城市、合肥都市圈节点城市的地位显著提升。六安中心城市首位度和承载能力明显提高。县城和集镇集聚能力日益增强。乡村振兴战略深入实施。城乡协调发展格局基本形成，人居环境更为改善。

**——民生保障更加有力。**城乡居民人均收入与经济发展同步增长。脱贫攻坚任务全面完成，贫困地区内生发展能力显著增强。实现更高质量和更加充分的就业，社会保障体系更加健全，社会事业全面发展，基本公共服务达到全省平均水平。

今后五年的主要任务是：

**第一，坚定不移贯彻新发展理念，着力建设现代化经济体系。**坚持质量第一、效益优先，推动经济发展质量变革、效率变革、动力变革，加快建设实体经济与科技创新、现代金融、人力资源协同发展的产业体系。深入实施绿色振兴战略，大力推进“一心一廊”“一谷一带”“一岭一库”建设，加快形成六大平台各具特色、多元统筹、竞相发展的格局。坚持把推进农业供给侧结构性改革作为主线，实施质量兴农战略，推进农业由增产导向转向提质导向，夯实农业生产能力基础，构建农村一二三产业融合发展体系。积极培育新型农业经营主体，推进“互联网+现代农业”，加快构建现代农业产业体系、生产体系、经营体系，不断提高农业创新力、竞争力和全要素生产率，推进农业农村现代化。加快新型工业化，聚焦高端制造、清洁能源、新能源汽车、新材料加工、电子信息、人工智能等产业，以开发区和工业园区为载体，大力发展战略性新兴产业。聚焦传统优势产业，实施新一轮技术改造专项行动，支持企业加快数字化、网络化、智能化改造，全面提高产品技术、工艺装备、能效环保和质量安全水平。开展质量提升行动，推进消费品工业增品种、提品质、创品牌，加快推动我市产业价值链向中高端延伸。充分发挥大别山生态资源和六安交通区位优势，突出发展现代物流、金融保险、电子商务等现代服务业，加快发展文化旅游、健康养老等产业。

**第二，坚持创新驱动发展，进一步增强发展动能。**深入实施创新驱动发展战略，加快建立以企业为主体、市场为导向、产学研深度融合的技术创新体系。强化平台要素供给，以六安大学科技园建设为抓手，布局建立一批低成本、便利化、开放式的众创空间，吸引中高级生产要素集聚，孵化一批“专精特新”创新型小微企业，培育一批具有创新能力的排头兵企业。统筹推进“三重一创”建设，加快建设一批重大新兴产业基地，扎实推进一批重大新兴工程，组织实施一批重大新兴产业专项，加快打造一批具有竞争力的战略性新兴产业集群。大力实施人才强市战略，积极引进战略科技人才、科技领军人才、青年科技人才和高水平创新团队。围绕使市场在资源配置中起决定性作用、更好发挥政府作用，突出抓好行政和经济体制、城乡发展体制、生态文明绿色发展体制、社会治理体制等重点领域和关键环节改革，争取实施更多国家级和省级改革试点。

**第三，坚持区域协调发展，全方位扩大对外开放。**做大做强做靓中心城市，加快东部产业新城、西部生态新区和绿色城南建设，扩大城市规模，完善城市功能。全面振兴县域经济，坚持特色化、差异化发展，找准县域发展路径，彰显特色、提质增效。支持舒城巩固发展融入合肥都市圈的六安桥头堡、合肥后花园地位。加快城镇发展，进一步完善通信网络、路网、管网、电网等基础设施，提高承载能力；加快重点镇建设，高品质建设一批特色小镇。深入推进乡村振兴战略，坚持农业农村优先发展，按照产业兴旺、生态宜居、乡风文明、治理有效、生活富裕的总要求，建立健全城乡融合发展体制机制和政策体系，统筹推进农村经济、政治、文化、社会和生态文明建设，加快推进乡村治理体系和治理能力现代化，让农业成为有奔头的产业，让农民成为有吸引力的职业，让农村成为安居乐业的美丽家园。全面扩大对外开放合作，积极参与长三角一体化，全面融入合肥都市圈，强化大别山区域合作，加快形成全面开放新格局。推进“单一窗口”通关服务体系推广应用，提升通关通检便利化水平。推进海关特殊监管区建设，打造对外开放新平台。培育对外贸易新业态新模式，大力发展加工贸易、服务贸易和跨境电商。

**第四，坚持社会主义核心价值体系，推动文化繁荣兴盛。**推进文化强市建设，坚持用中国梦和社会主义核心价值观凝聚共识、汇聚力量，建设“诚信六安”，打造“好人六安”；持续深化精神文明创建，争创第六届全国文明城市。充分挖掘宣传弘扬六安红色精神、淠史杭精神、大别山精神，加强社会公德、职业道德、家庭美德和个人品德建设。加快发展文化事业和文化产业，深化文化体制改革，完善文化管理体制，培育一批骨干文化企业。深入实施文化惠民工程，完善公共文化服务体系。加强文物保护利用和非物质文化遗产保护传承，支持优秀文化产品创作生产。组织更多高水平体育赛事，加快体育事业和体育产业融合发展。丰富群众性文化体育活动，持续打造大别山（六安）山水文化旅游节、“社区一家亲”、“毅行六安”等公共文化活动品牌。

**第五，坚持在发展中保障和改善民生，不断满足人民美好生活需要。**深入实施精准扶贫精准脱贫方略，把提高脱贫质量放在首位，注重扶贫同扶志、扶智相结合，大力推进脱贫攻坚“十大工程”，确保脱贫攻坚目标如期实现。以深山区、库区和沿淮行蓄洪区为主战场，加大投入力度，补齐发展短板。着力培育内生发展能力，构建脱贫长效机制，确保稳定脱贫不返贫。加强基础设施建设。完善铁路、高速公路、国省干线网络，提升县乡公路建设管理水平，努力实现“县县通铁路通高速、市县一级路、乡乡二级路、村道五米五、乡村通公交”的目标。深入实施“水利六安”战略，完善河湖管护体系，实现淮河安澜、河湖健康、人水和谐。加快电网、信息、物流等基础设施网络建设。坚持尽力而为、量力而行，聚焦“七有”领域，每年实施一批群众急需、普惠大众的市级民生工程。加快教育现代化，统筹各级各类教育发展，办好人民满意的教育。实施就业优先战略和积极就业政策，鼓励创业带动就业，实现更高质量和更充分的就业。全面建成覆盖全民、城乡统筹、权责清晰、保障适度、可持续的多层次社会保障体系。加快建立多主体供给、多渠道保障、租购并举的住房制度。编制实施“健康六安2030”规划，推进医养结合，发展康养产业。全面建立基本医疗卫生制度、医疗保障制度、优质高效医疗卫生服务体系和现代医院管理制度。深化平安六安建设，完善社会矛盾纠纷多元化解机制，提高社会治理社会化、法治化、智能化、专业化水平。健全公共安全体系，全面加强社会治安、生产安全、食品药品安全工作，让社会持续安定、人民永续安康。

**第六，坚持人与自然和谐共生，打造生态文明建设六安样板。**强化主体功能区定位，科学划分空间开发格局，强化土地节约集约利用。坚持全民共治、源头防治，大力实施环境保护“五个一”行动，坚决打好污染防治攻坚战。深入实施蓝天工程、碧水工程和净土行动，严格落实“河长制”，全面推行“林长制”。突出抓好森林、湿地、流域、农田、城市“五大生态”建设，统筹山水林田湖系统保护和修复，不断提升生态系统质量和稳定性。积极开展生态创建，争创六安大别山国家公园、大别山国家湿地公园和国家生态园林城市。完善生态文明制度体系，改革生态环境监管体制。健全大别山区水环境生态补偿机制，探索建立大别山水权制度。完善环境治理和生态修复制度，开展领导干部自然资源资产离任审计，实行最严格的源头预防、过程控制、损害赔偿、责任追究制度，坚决制止和惩处破坏生态环境行为，确保六安生态优势持续长久、青山绿水永葆美丽。

三、全面贯彻党的十九大精神，奋力实现各项工作新开局

今年是贯彻党的十九大精神的开局之年，是六安站在新起点、迈上新征程的起步之年，也是实施“十三五”规划承上启下的关键一年。按照市委四届六次全会部署，今年经济社会发展的主要预期目标是，地区生产总值增长8%左右，财政收入增长10%，规模以上工业增加值增长8%，固定资产投资增长10%以上，社会消费品零售总额增长11%，城镇、农村常住居民人均可支配收入分别增长8%、9%，城镇新增就业2.8万人，城镇登记失业率控制在4.2%以内。

重点做好八个方面工作：

**（一）全力促进经济平稳健康发展。**

**扩大有效投入。**落实“四督四保”和“五项机制”，确保全年新开工亿元以上项目200个、完成投资800亿元以上。紧抓产业投入，加快美盈森工业4.0智慧工厂、金安氢能源汽车产业园、应流航空产业园、沃特玛创新联盟舒城新能源汽车产业园、迎驾野岭产业园、森德自动化高端装备制造、叶集绿色板材工业园等项目建设，推动霍邱钢厂复工投产。加快交通基础设施建设投入，推进合安高铁舒城段、合阜高速霍邱段建设以及六武高速六安段、京台高速舒城段“四改八”工程，新改建国省干线公路642公里。推进淮河流域综合治理，完成淮干一般堤防加固工程建设。加大中央预算内资金争取力度，发挥政府资金引导作用，积极推广运用政府和社会资本合作（PPP）模式。大力推进招商引资，确保到位内资增长10%、外资增长7%；突出产业链招商，强化以商招商，推进合作招商。充分利用IPO绿色通道政策，开展精准招商。

**促进消费和进出口。**开展放心消费创建活动，提升旅游休闲、文化体育、健康养老等服务消费水平，引导新能源汽车等绿色产品消费，培育壮大智能家居等新兴信息消费。推进城区农贸市场改造升级。培育发展商贸流通主体，力争新增限上商贸企业100家以上。大力拓展对外贸易，扩大机电、汽车零部件、农产品等产品出口。积极发展进口贸易，支持企业扩大先进技术、设备和关键零部件进口。

**加大财税金融支持实体经济力度。**常态化推进“暖企”行动、“四送一服”双千工程。进一步壮大市产投公司、扩大产业基金规模，引导发展各类股权投资。全面落实降成本各项措施，持续降低企业制度性交易成本。大力发展普惠金融，改善农村、中小微企业等金融服务，扩大政策性融资担保、“税融通”等新型融资模式。强化政银担企对接，力争新增贷款180亿元以上。继续发掘和培育优质上市挂牌后备资源，新增上市报审企业1家、上市报备企业2家、“新三板”挂牌企业3家，力争直接融资55.5亿元以上。打好防范化解重大风险攻坚战，妥善处理政府债务，加快存量政府债务置换，合理确定发债标准和规模；健全金融风险监测预警和应急处置机制，坚决打击违法违规金融活动，守住不发生系统性金融风险底线。

**（二）加快建设现代化产业体系。**

**强力推进农业现代化。**深入实施“特色产业基地、农产品加工、主体培育、新产业新业态、品牌农业”五大提升工程，打造粮油、茶叶、蔬菜、林果等特色产业集群。做大做强国家和省级农业产业化龙头企业，新培育一批10亿元以上的甲级队。构建现代农业经营体系，大力培育新型农业经营主体，发展家庭农场联盟、合作社联合社和农业产业化联合体，加快建设知识型、技能型、创新型的新农民队伍。健全农业社会化服务体系，建设综合性全程农事服务中心（农事大院）20个。发展壮大村级集体经济，培养造就一支懂农业、爱农村、爱农民的“三农”工作队伍。

**大力发展先进制造业。**加快实施重点产业规划，培育壮大六大主导产业，打造九大产业集群，力争主导产业增加值占全部工业比重达70%以上。深入实施“中国制造2025”六安篇，大力发展新一代电子信息、装备制造及核心零部件、新能源汽车等先进制造业，力争其首位度超过20%以上。改造提升传统产业，实施新一轮技术改造，推进一批新技术应用、新产品开发、新模式嵌入和“机器换人”项目，力争技改投资增长15%以上。实施“专精特新”企业培育工程，打造一批行业“单项冠军”和“配套专家”。

**加快发展现代服务业。**继续实施旅游产业重点项目三年行动计划，加快大别山国家风景道建设，积极创建大别山主峰5A景区、大别山（六安）湿地和六安茶谷核心区国家旅游度假区。加快乡村旅游集聚区建设，推进旅游与文化、生态、健康等产业深度融合。加快综合物流园、物流中心和配送节点建设，促进现代物流业集聚发展。大力发展技术服务、工业设计、人力资源培训等生产性服务业。加快电子商务进农村，培育平台经济、分享经济、体验经济等新业态。

**增强创新能力。**加快六安大学科技园二期、裕安双创大厦等创新服务平台建设，力争新组建产业技术创新战略联盟1-2个、新设立院士工作站2-3个。大力发展战略性新兴产业，加快省市级基地和重大试验工程、重大专项建设。支持地方、企业与高校院所联合组建产业技术创新战略联盟和新型科技研发机构，推动跨行业、跨领域协同创新。实施高新技术企业培育工程，力争实现产值400亿元，新认定高新技术企业20家。全面落实市深化人才发展体制机制改革的实施意见，加快人才引进、培养和流动。加强知识产权创造、运用和保护，新增授权发明专利180件。

**加快开发园区转型升级。**推进开发园区整合优化，明晰产业定位，实现错位发展。高标准建设园区基础设施、标准化厂房、“邻里中心”，提升承载能力。加大“僵尸”企业和闲置资产、土地处置力度，实施循环化改造，推进节约集约发展。做大做强六安经济技术开发区，支持省级开发区争创国家级开发区和省级高新区。

**（三）坚决打赢脱贫攻坚年度战役。**

**以绣花功夫实施脱贫攻坚十大工程。**全面推广“四带一自”产业扶贫模式，深入推进特色种养业、旅游、互联网、光伏、新型经营主体、资产收益+脱贫，健全产业扶贫利益联结机制。深入开展智力扶贫，实现贫困家庭“雨露计划”和建档立卡贫困户学生资助全覆盖。巩固健康脱贫“351”“180”政策成果，落实农村低保标准和扶贫标准“两线合一”。完成易地扶贫搬迁3229户、贫困户危房改造1.3万户。规范开展扶贫小额信贷，积极开发保险扶贫产品，推进邮政电商精准扶贫。

**加大薄弱环节和深度贫困地区补短板力度。**整合各类帮扶资源，集中力量攻坚，确保新增脱贫攻坚资金、项目、措施主要集中于深山区、库区、沿淮行蓄洪区等深度贫困地区。加快推进移民搬迁、庄台建设、环境整治和适应性发展。围绕贫困地区文化、教育、医疗、就业、住房等领域，加快补短板，推进基本公共服务均等化。

**压紧压实各方责任。**健全脱真贫、真脱贫工作机制，强化一把手负责制，坚决做到“七个不落、一个不少”，确保舒城县、金安区、裕安区摘帽，185个贫困村出列，13万以上贫困人口脱贫。完善防范返贫机制，加强扶贫对象动态管理，落实脱贫后续帮扶措施。深入开展结对帮扶，健全“单位包村、干部包户”和驻村扶贫工作队管理制度。从严落实激励奖惩措施，严肃处理扶贫领域违法违纪案件。继续开展“扶贫日”、扶贫志愿者和“百企帮百村”活动。

**（四）积极实施乡村振兴战略。**

**增加绿色优质农产品供给**。加快粮食生产功能区和重要农产品生产保护区建设，稳定粮食产能，扩大绿色、特色农产品供给。推进霍山石斛、灵芝、断血流、茯苓等“十大皖药”示范基地建设，打造皖西白鹅、皖西麻黄鸡等特色畜牧业。扩大高效优质经济作物比重，推广设施栽培、生态养殖、立体种养等技术模式。加强农业科技研发和创新能力建设，健全特色农产品质量标准化体系。

**加快补齐农村基础设施建设短板。**加大交通、能源、水利、信息、环保等重点领域建设投入力度，加快推动城镇基础设施向农村延伸。投资13.7亿元，实施农村公路2660公里，推进“四好”农村路建设，加快农村公路“建管养运”一体化发展。实施农田水利“最后一公里”规划，综合整治小型农田灌排区86万亩，实施8条中小河流治理、96座小水库除险加固。推进农村饮水安全工程，提高农村自来水普及率、集中供水率和水质达标率。实施新一轮农村电网改造，加快水电供区电网移交与改造，推进城乡电网融合发展。

**提升美丽乡村品质。**持续加强美丽乡村、农村社区建设，加快乡镇政府驻地整治，继续推进美丽乡村中心村及自然村环境整治。深入推进农村垃圾、污水、厕所专项整治“三大革命”，启动“生态宜居新村”建设。开展村民自治试点，推动移风易俗，培育文明乡风、良好家风、淳朴民风，不断提高乡村社会文明程度。

**深入推进农村综合改革。**巩固扩大农村土地确权登记颁证成果，推进农村土地“三权”分置改革，加快农村产权交易市场建设。深化农村集体产权制度改革，扩大农村“三变”改革试点。完成金寨县农村土地制度“三项改革”和全国农村集体产权制度改革试点。巩固完善国有林场和集体林权制度配套改革。推进农业保险试点改革。

**（五）深入推进重点领域改革。**

**深化机构和行政体制改革。**优化机构设置，开展编制“周转池”制度试点。扎实推进监察体制改革试点。深化“放管服”改革，健全提升权责清单制度体系，全面实施市场准入负面清单制度，推进行政审批标准化建设。深化商事制度改革，全面实施“多证合一”、“证照分离”，加快企业登记全程电子化和电子营业执照应用。推进“互联网+政务服务”，形成全市政务服务“一张网”。推进事业单位分类改革，巩固完善市直生产经营服务类事业单位改革。

**加快财税金融改革。**进一步规范财政转移支付管理，完善市区财政管理体制。深化预算制度改革，着力构建规范透明、标准科学、约束有力的预算制度。完善国有资产监管体制机制，完成党政机关、事业单位与所属企业脱钩改革，加快市国资管理运营公司组建步伐。推进政府性投融资平台转型发展。加强地方金融体系建设，规范发展新型金融组织。加快信用体系建设步伐，完善金融监管体制机制，改善金融服务环境。

**深化其他重点领域改革。**深化文化体制改革，鼓励和引导非公有制文化企业发展。推进教育改革，继续实施普通高中学业水平考试和综合素质评价。深化医药卫生体制改革，建立健全现代医院管理、基本医疗卫生和医疗保障制度，推进医联体建设，实现县域医共体建设全覆盖。改革审计管理体制，保障审计监督全覆盖。推进机关事业单位基本养老保险制度改革。加快事业单位公车改革。

**（六）加快新型城镇化建设。**

**做大做强中心城市。**统筹推进东部产业新城、西部生态新区、绿色城南建设，着力防治交通拥堵、环境污染、产业空心化、文化生活贫乏等“城市病”。启动新一轮中心城市总体规划修编和空间规划编制。继续加大中心城区重点工程建设，实施佛子岭路改造、城区公交线网优化、棚户区改造等115个项目。持续推进海绵城市、智慧城市建设，提升城市品质。以争创全国文明城市为抓手，全面推进城管综合执法，深入开展市容市貌、道路交通秩序整治，建立健全长效机制。加强房地产市场调控，完善开发用地和商品房供应管理，推进非住宅商品房去库存，培育发展住房租赁市场，促进房地产市场持续健康发展。

**加强县城和集镇建设。**推动霍山县、舒城县“县改市”对标创建，支持霍邱县城建设中等城市。大力推进“两治三改”，统筹推进路网、管网、电网等基础设施建设，完善教育、文化、医疗等公共服务设施，着力改善人居环境，加快打造一批特色小城镇。推进公共交通优先发展示范市建设，完成金安区、裕安区、叶集区城乡公交一体化。

**大力推进区域协调发展。**推进合六一体化，加快S366合六南通道建设，争取合六市郊铁路开工，推动合六工业走廊共建，推进丰乐河、杭埠河等合作治理。加快大别山革命老区振兴，实施一批基础设施、社会事业、生态环境、扶贫开发工程和项目，提升自我发展能力。积极推进区域合作共建，提升合肥高新区霍邱现代产业园、舒城包河现代产业园发展水平，推进合肥经开区与霍山共建高端装备合作园区。

**（七）推进生态文明建设**

**加快绿色发展平台建设。**全面推进合六叶工业走廊暨金叶一体化，积极谋划合六产业带。加快六安茶谷、淮河淠河生态经济带“三产”融合发展，打造一批主题公园、林果庄园和乡村田园综合体，发展一批观光休闲、科普教育、自驾车（房车）营地、养生保健等旅游休闲基地。推进“江淮果岭”建设，大力发展以桃、猕猴桃、葡萄为主的林果产业和以山核桃、油茶为主的木本油料产业。规划建设“西山药库”，推动优势特色中药材种植、储存、加工和销售一体化发展。

**打好污染防治攻坚战。**持续抓好中央和省环保督察反馈问题整改，着力解决突出环境问题。深入推进大气、水、土壤污染防治，确保PM 10、PM2.5达到国家和省考核要求；继续实施重点河流水污染防治项目，推进城市黑臭水体整治，强化水源地环境保护；启动土壤污染详查，加强污染地块和农用地土壤环境管理。严格执行排污许可制度，探索实施环境信用联合激励和惩戒措施。

**加强生态保护。**严守生态保护红线，划定城镇开发边界。巩固大别山水环境生态补偿试点成果，认真实施《六安市饮用水水源环境保护条例》，深入开展山水林田湖综合治理和生态修复，治理水土流失面积120平方公里；推进林业增绿增效行动，新增人工造林6.2万亩、退化林修复14万亩，全面停止天然林商业采伐。

**（八）提高保障和改善民生水平。**

**巩固提升民生工程。**在实施好省33项民生工程的基础上，实施市区城乡公交一体化、城区停车场建设、社区康养中心3项市级项目，加快建立政府主导、多方参与的民生工程资金筹措机制。积极探索创新民生工程建后管养模式，推进民生工程持久发挥效益。

**加强就业和社会保障工作。**实施更加积极的就业政策，着力构建和谐劳动关系，保障农民工工资按时足额支付。全面落实社会保障和社会救助政策，突出抓好困难群众基本生活保障。完善城乡居民基本医疗保险和大病保险制度，全面实施全民参保计划。调整机关事业单位工作人员基本工资标准，提高企业退休职工基础养老金、城乡低保对象补助水平。积极推动养老事业多元化、多样化发展，推进居家养老服务。

**加快教育医疗卫生事业发展。**认真实施学前教育第三个三年行动计划，加强学前教育监管。完成六安城区义务教育段公办学校第二个三年行动建设计划，有序推进新一轮普通高中布局调整和课程改革。支持安师大叶集分校建设。加强基层医疗卫生服务体系和全科医生队伍建设，全面推进家庭医生签约服务，推进乡村卫生服务一体化，实施名医、名科、名院、名药工程，推进中医药事业发展。

**推动文化体育发展。**全面提升文化产品和服务供给能力，完善现代化公共文化服务体系，推进市体育中心等重点项目建设，加快基层综合性文化服务中心建设。深入开展全民健身运动，发展群众体育和竞技体育。组织开展改革开放40周年、“淠史杭工程”开工60周年纪念活动。

**打造共建共治共享的社会治理格局。**深入推进“七五”普法，加强困难群众法律援助。健全基层社会治理体系，推进智慧社区建设。继续开展“无越级上访乡镇”创建活动，推动信访工作重心下移、关口前移。深入开展安全生产“铸安”行动，坚决遏制较大及其以上安全生产事故。强化食品药品安全监管，创建省食品药品安全城市。加强社会治安防控体系建设，依法打击和惩治各类违法犯罪活动。加快应急平台和应急专业队伍建设，提升防灾减灾救灾能力。加强国防动员和国防教育，推进军民深度融合发展。统筹做好统计、物价、广播电视、新闻出版、地方志、气象、地震、对台、档案、邮政、外事侨务、老龄、全民科学素质、妇女儿童、残疾人等工作。

四、全面增强本领，推动政府工作再上新台阶

新时代要有新作为，新作为需要新本领。我们要以党的十九大精神为统领，不断加强政府自身建设，切实提高为民服务、推动发展的能力和水平。

**一要始终做到忠诚看齐。**自觉用习近平新时代中国特色社会主义思想武装头脑、指导实践、推动工作。牢固树立“四个意识”，增强“四个自信”，坚定维护以习近平同志为核心的党中央权威和集中统一领导。推进“两学一做”学习教育常态化制度化，认真开展“不忘初心、牢记使命”主题教育，强化理论武装。坚持知行合一、学以致用，切实把党的十九大精神贯彻落实到政府工作各方面和经济社会发展全过程，确保中央和省、市委各项决策部署落到实处。

**二要提升科学履职能力。**注重学习新知识、把握新规律，全面增强学习本领、政治领导本领、改革创新本领、科学发展本领、依法执政本领、群众工作本领、狠抓落实本领、驾驭风险本领。加快构建决策科学、执行坚决、监督有力的权力运行机制，推进政府治理体系和治理能力现代化。提升行政决策水平，落实利益相关方、公众代表、专家、媒体等列席政府有关会议制度，增强决策透明度、社会认同度。

**三要深入推进依法行政。**牢固树立法治意识，自觉运用法治思维和法治方式深化改革、推动发展、化解矛盾、维护稳定。严格规范执法行为，全面落实行政处罚裁量权基准制度，推动重大行政执法决定法制审核全覆盖。坚持以公开为常态、不公开为例外，推进决策公开、执行公开、管理公开、服务公开和结果公开。增强人大意识和人大代表主体意识，自觉接受党内监督、人大监督和政协民主监督，认真办理人大代表议案、建议和政协委员提案，提高办理成效。广泛听取各民主党派、无党派人士意见，发挥工会、共青团、妇联等人民团体重要作用，主动接受司法监督、群众监督、舆论监督，加强政府内部层级监督和专门监督，让政府权力在阳光下运行。

**四要提高为民服务效能。**进一步改进工作作风，坚持“严”字当头、“实”字为先、“公”字为本，狠抓工作落实，增强政府执行力。健全为民服务体系，创新服务模式，健全运转机制，着力打通服务群众“最后一公里”。加强乡镇政府能力建设，提升为民服务水平。完善政绩考核评价体系，健全激励和容错纠错机制。继续推进“电视问政”“万人评科长”“社会评窗口”工作，大力整治“庸、懒、散”行为，切实提高行政效能。

**五要持之以恒正风肃纪。**坚定不移落实全面从严治党主体责任，持续提升政府系统从严管党治党水平。认真落实党风廉政建设责任制，进一步落实中央八项规定及实施细则和省、市委有关规定，盯紧“四风”新变异新动向，重点解决形式主义、官僚主义问题。规范招投标秩序，严格公共资源交易监督管理。进一步规范市直单位财务管理，严肃财经纪律，强化审计监督。坚持惩治腐败无禁区、全覆盖、零容忍，支持纪检监察机关依法依规履行职责，坚决查处各类违纪违法案件。

各位代表！新时代开启新征程，新时代赋予新使命。让我们更加紧密地团结在以习近平同志为核心的党中央周围，在市委的坚强领导下，凝心聚力，奋发图强，确保完成今年经济社会发展目标任务，为如期全面建成小康社会、开创新时代幸福六安建设新局面而不懈奋斗！
